# Photodynamic Therapy: Current Trends and Potential Future Role in the Treatment of Bladder Cancer

**DOI:** 10.3390/ijms25020960

**Published:** 2024-01-12

**Authors:** Maxim Kochergin, Omar Fahmy, Anastasios Asimakopoulos, Gerit Theil, Kathleen Zietz, Johanna Bialek, Eugenio Tiberi, Georgios Gakis

**Affiliations:** 1 Department of Urology and Neurourology, BG Unfallkrankenhaus Berlin, 12683 Berlin, Germany; max.kochergin84@gmail.com (M.K.); eugenio.tiberi@ukb.de (E.T.); 2 Department of Urology, Universiti Putra Malaysia (UPM), Serdang 43400, Malaysia; docomar82@gmail.com; 3 Urology Unit, Fondazione PTV Policlinico Tor Vergata, 00133 Rome, Italy; tasospao2003@yahoo.com; 4 University Clinic and Polyclinic of Urology, University Hospital of Halle, Martin-Luther University Halle-Wittenberg, 06099 Halle, Germany; gerit.theil@uk-halle.de (G.T.); kathleen.zietz@uk-halle.de (K.Z.); johanna.bialek@uk-halle.de (J.B.)

**Keywords:** photodynamic therapy, bladder cancer, urothelial carcinoma, in vivo, in vitro, cell line, animal model

## Abstract

Bladder cancer (BC) is the 10th most common cancer in the world. The therapeutic spectrum of BC is broad and is constantly expanding. Despite the wide clinical use of photodynamic diagnosis (PTD) for BC, PDT has not been sufficiently investigated in the treatment landscape of BC. We performed an online search of the PubMed database using these keywords: *photodynamic therapy*, *bladder cancer*, *urothelial carcinoma*, in vivo, in vitro, *cell line*, *animal model.* Reviews, case reports, and articles devoted to photodynamic diagnostics and the photodynamic therapy of tumors other than urothelial carcinoma were excluded. Of a total of 695 publications, we selected 20 articles with clinical data, 34 articles on in vivo PDT, and 106 articles on in vitro data. The results presented in animal models highlight the potential use of PDT in the neoadjuvant or adjuvant setting to reduce local recurrence in the bladder and upper urinary tracts. Possible regimens include the combination of PDT with intravesical chemotherapy for improved local tumor control or the integration of vascular-targeted PDT in combination with modern systemic drugs in order to boost local response. We summarize available evidence on the preclinical and clinical application of PDT for urothelial carcinoma in order to explain the current trends and future perspectives.

## 1. Introduction

Bladder cancer (BC) is the 10th most common cancer in the world and the 13th cause of cancer-related deaths worldwide. Considering the European continent alone, BC ranks seventh in the incidence scale and fifth in the mortality rate [1]. The standard spectrum of therapy for BC is broad and is constantly expanding due to new scientific developments and new insights into tumor biology. In addition to surgical therapy and established local and systemic treatment modalities, such as chemotherapy and immunotherapy [2], there exists therapeutic modalities that are currently outside the scope of clinicians treating patients with urothelial carcinoma (UC). Despite substantial experience in other tumor entities and the wide clinical use of photodynamic diagnosis (PDD) for the transurethral resection of bladder tumor (TURBT), the potential of photodynamic therapy (PDT) for the treatment of UC has not been thoroughly investigated [3,4,5,6,7,8,9].

In PDD, following instillation with a photosensitizer, the bladder is exposed to light of a specific wavelength. The photosensitizer accumulates in urothelial cells and causes the emission of red light. The result is an image of red fluorescent tumor tissue surrounded by blue healthy tissue. On the other hand, in PDT, the role of the photosensitizer is not to visualize neoplastic cells, but to induce apoptotic effects [10]. PDT has been investigated in the treatment of BC for over three decades. Prout et al. conducted a collaborative study to evaluate the efficacy of this form of therapy in the treatment of superficial BC. They used a hematoporphyrin derivative as photosensitizer and showed that PDT is promising in treating superficial BC [11]. The aim of this review is to summarize the available preclinical and clinical data on PDT in order to explain the current trends and future perspectives of this therapeutic modality in UC.

## 2. Materials and Methods

Two authors (MK, ET) performed a systematic, independent online search of the PubMed database as of September 2022. The following keywords were used: *photodynamic therapy*, *bladder cancer*, *urothelial carcinoma*, in vivo, in vitro, *cell line*, *animal model.* Reviews, case reports, and articles which primarily dealt with PDD and PDT of tumors other than UC were excluded. Articles were selected by title and then by abstract. Then, the appropriate articles were subjected to full-text evaluation, and the bibliography was searched. Finally, an isolated selection was made according to mechanisms, in vitro, in vivo, and clinical data. Of a total of 695 publications that appeared in the search for PDT in BC without a time frame, we selected 20 articles for clinical data, 34 articles for in vivo PDT, and 106 articles for in vitro data analysis.

## 3. Results

### 3.1. Mechanisms of Photosensitization

The principle of PDD and PDT is based on the exposure of tumor cells to a photosensitizer (PS). This is absorbed by the tumor cells to a greater extent than by healthy cells. Subsequently, in the case of PDD, illumination with a light source of the appropriate wavelength leads to better identification of the tumor in the case of PDD and to the death of tumor cells in the case of PDT [12].

### 3.2. Photosensitizers in Bladder Cancer

For this review, we focus on PS that are most commonly used or have the greatest importance in the treatment of BC.

#### 3.2.1. ALA

5-ALA (aminolevulinic acid) is a precursor of protoporphyrin IX, which functions as the actual PS, and, otherwise, a precursor of hemoglobin and chlorophyll. The endogenous production of 5-ALA occurs from glycine and succinyl-CoA. Exogenous ALA can be administered topically. After the formation of a precursor in the cell cytoplasm, it is transported via the ATP-binding cassette of subfamily B, member 6 (ABCB6), to the mitochondria to form protoporphyrin IX, which is converted to heme after the incorporation of iron catalyzed by ferrochelatase [13].

Protoporphyrin IX accumulates in cancer cells, and after light exposure with an appropriate wavelength (green or red light), the reactive oxygen species lead to cancer cell damage. The accumulation of protoporphyrin IX by healthy cells is much lower, which explains the selectivity in its effect against cancer cells. Heme is important for the further production of ATP in the aerobic metabolic pathway. The metabolism of the tumor cell differs from that of the normal cell and is based on oxygen-independent glycolysis for ATP production, which is known as the Warburg effect. The inactivation of ferrochelatase and ABCG2 in cancer cells leads, on the one hand, to impaired heme production and the impaired excretion of protoporphirin IX. On the other hand, the activation of the synthetic enzyme 5-ALA and peptide transporter 1 in tumor cells leads to the increased accumulation of 5-ALA in the mitochondria of tumor cells. Specifically for UC cells, the concentration of protoporphirin IX was shown to be 17-fold higher than in healthy urothelium. These properties of 5-ALA determine its suitability for PDD and PDT.

When exposed to visible blue light in the range of 375–445 nm, protoporphyrin IX, which is enriched in tumor cells, emits red fluorescent light in the range of 600–740 nm. These conditions are used for PDD in BC [13].

When irradiated with a longer-wavelength-light in the green (450–580 nm) and red (600–740 nm) spectra, the accumulated protoporphyrin IX leads to energy release and the formation of reactive oxygen species by changing its state, with subsequent damage to cell mitochondria and cell death by apoptosis. These cells undergo further phagocytosis, and because the inflammatory effect caused by necrosis is absent, there is no relevant collateral damage to adjacent structures [13].

#### 3.2.2. Hypericin

Hypericin is an anthraquinone derivative derived from St. John’s wort (Hypericum perforatum). The accumulation of hypericin in the cell occurs in the membranes of the nuclear envelope, endoplasmic reticulum, Golgi complex, and mitochondria [14]. Light absorption occurs at a wavelength of 514–593 nm. The following mechanisms of action have been proposed in the literature: (1) generation of active oxygen species as a result of photoactivation by hypericin; (2) rapid loss of calcium stores as a result of the accumulation of hypericin in the endoplasmic reticulum membrane with subsequent activation of apoptosis; (3) inhibition of protein kinase C and some other growth factors, leading to the increased peroxidation of membrane lipids; (4) release of cytochrome C from mitochondria, leading to an increase in the activity of procaspase-9 and 3 and PARP (poly-ADP-ribose polymerase) cleavage, resulting in mitochondrial damage and subsequent apoptosis [14].

#### 3.2.3. Chlorophyllin

Chlorophyllin is produced from chlorophyll derived from chloroplasts of plants or from cyanobacteria. Chlorophyllin is accumulated in the mitochondria and lysosomes of the cell. Autophagy and apoptosis are held responsible as the main mechanism of action. Light absorption occurs at a wavelength of 600–670 nm and leads to a relevant increase in reactive oxygen species, accompanied by a decrease in the activity of superoxide dismutase. It is water-soluble, cleavable, low-toxic, and rapidly excreted from the body [14].

#### 3.2.4. Palladium Bacteriochlorophyll Derivatives

The most commonly described agent in this group, WST11 (Tookad© solubleH), is a laser-activated vaso-occlusive agent that selectively persists in the bloodstream and is rapidly excreted by the liver and kidneys. The photoactivation of WST11 results in the formation of reactive oxygen species, which triggers reactions that lead to vascular damage and plugging with subsequent necrosis of tumor cells 48 h after treatment. To achieve this selectivity, the optical fiber must be introduced into the tumor. This mechanism differs from the classical PDT effect at the cellular level. This type of PDT has been referred to in the literature as “vascular targeted photodynamic therapy” (VTP). The efficacy of WST11-VTP has even been demonstrated in phase II clinical trials for the treatment of localized prostate cancer [15]. This type of PDT induces the proliferation of dendritic cells and macrophages. The ability to infiltrate the tumor was demonstrated for Mac2- and CD3+-stained cells and resulted in further activation of lymphoid cells. This type of PDT appears to elicit a long-term immune response as evidenced by increased numbers of T cells at all foci and a specific increase in CD8+ and active CD4+ T cells long after treatment [15].

#### 3.2.5. Chlorins

Chlorins (dihydroporphyrins) are efficient porphyrin-derived PSs activated by near-infrared light. The most widely described PS of this group include m-tetrahydroxyphenyl chlorin (temoporfin or Foscan), benzoporphyrin (verteporfin), and radachlorin (mixture of sodium salts of chlorin e6, chlorin p6, and purpurin) [16].

#### 3.2.6. Tetrahydroporphyrin Tetratosylate (THPTS)

THPTS is a hydrophilic cationic PS that is thought to accumulate in lysosomes. Uptake into cells is thought to occur by pinocytosis. Accumulation in cells was observed regardless of their type or metabolic activity, but accumulation in lysosomes was detected only in tumor cells. The toxic effect on the tumor cell occurs either via the release of lysosomal enzymes or via the release of PS from lysosomes after light exposure and damage to the other cell components. An increase in the activity of caspases 3 and 9 was observed. The photoactivation of specific genes leading to growth arrest and activation of apoptosis was also described, as well as the upregulation of HSP105 and the increased concentration of mRNA for GADD45α leading to the activation of MEKK4 and the subsequent activation of the apoptotic protein p38. Maximum absorption occurs in the near-infrared region (760 nm), which allows for tissue penetration of up to 15 mm [17].

### 3.3. In Vitro Studies

The effects of PDT have been extensively studied in vitro in animal- and human-bladder cancer cell cultures exposed to a photosensitizer with light activation. In these studies, relevant aspects of the mechanism of action of different PSs leading to cell death were investigated, and different types of light exposure were used. In vitro studies represented the majority of publications (>100 similar publications for different PSs), so that the detailed analysis would be beyond the scope of this review. For this reason, we presented the key features of the recent in vitro studies.

Zhang et al. [18] presented a new porphirin-derived photosensitizer (TPPP) with promising results in human BC T24 cells. After irradiation with a 650 nm laser, cells injected with TPPP showed clear signs of necrosis after 3 h [18]. Pereira et al. [19] demonstrated an enhanced therapeutic effect of the combination of chlorin and galactodendritic units (ChGal8) in UM-UC-3 and HT1376 BC cells after one and two cycles of irradiation, respectively; a second irradiation appeared to increase mithocondrial permeability to ChGal8 through GLUT-1 [19]. Lin and colleagues sought to improve PDT effects by modulating tumor hypoxia and used in vitro and in vivo mice models of orthotopic BC to investigate the effect of Chlore6-based PS enhanced with oxygen-producing nanoparticles (HSA-MnO2-Ce6 NPs: HSA—human serum albumin, Ce6—Chlore6, NPs—nanoparticles). The authors observed a significant increase in oxygen concentration in both in vitro and in vivo models and reported a markedly increased therapeutic efficacy of PDT, which resulted in prolonged survival of mice [20]. Previous in vitro research has also investigated factors responsible for tumor cell survival. A study by Pagliarone et al. [21] investigated the role of the heat shock protein HSPA9 (mortalin) against oxidative shock induced by PDT in the UC cell line MB49: mortalin was found to be highly expressed in MB49, and its inhibition enhanced oxidative damage to cancer cells upon exposure to PDT [21]. Stavropoulos et al. [22] described the polar methanol fraction of Hypericum perforatum L. extract as a PS for PDD and PDT on the two human BC cells’ lines T24 (high-grade metastatic cancer) and RT4 (low-grade primary papillary cancer). A PS at a concentration of 60 μg/mL caused a high cytotoxic rate (80–86%) in both cell lines and did not induce cell death at all at a lower concentration of 20 μg/mL. Photofrin showed a lower cytotoxic effect of 77% in T24 cells and 9% in RT4 cells [22]. Another attempt to increase the efficacy of PDT was described by Bhuvaneswari et al. who used hypericin in combination with Erbitux (an angiogenesis inhibitor), which acts on the epidermal growth factor receptor (EGFR) in human bladder cancer cells. The results showed that the combination of Erbitux with hypericin had a significantly higher inhibitory effect [23].

Photosensitizer-loaded nano particles were investigated as intravesical drugs to improve PDT performance in treating BC. The advantage of intravesical therapy is mainly to reduce the systemic side effects and increase the exposure of bladder cancer cells. Yet it is limited by the fast washout of the drug with a voiding, dilutional effect of the urine [24]. Phosphonic acid-containing groups are inhibitors of urokinase plasminogen, a key enzyme in metastasis, and cell invasion has shown higher accumulation in bladder cancer epithelial cells UM-UC-3 than ARPE19 cells, higher ROS production, and IC50 values of 1.154–1.476 μM for urokinase plasminogen inhibition. 

These in vitro studies show that PDT has been improved in many ways such as improved targeting of cancer cells, route of administration, formulation, and light conditions.

### 3.4. In Vivo Studies

One of the first in vivo studies was published in 1987 by Morgan et al. [25]. Fischer rats with transplanted urothelial tumors were irradiated with red light (599 nm) after treatment with porphyrin derivatives and developed tumor necrosis 24 h after treatment, whereas no necrosis occurred in the control group, demonstrating the selectivity of PS [25]. Two years later, Bellnier et al. published an in vivo study on Photofrin II in a mouse model with and without tumor. They reported the distribution of PS in all organs and showed that the major route of excretion of Photofrin II is feces [26].

In 1992, Han et al. reported PDT with a hematoporphirin derivative on human-bladder tumor cells (BL-17) implanted subcutaneously in immunodeficient Balb/c mice. PDT resulted in tumor cure in 71% of the animals. The authors demonstrated that the effect was due to the combination of PS and light exposure. It did not depend on the type of laser used [27]. In 1996, Post et al. compared three different PSs in a mouse model for their ability to produce functional bladder injury [28]. The PS used were photofrin, m-THPC, and bacteriochlorin. The most intense histologic changes after all three photosensitizers were submucosal edema and vasodilatation with epithelial denudation. Recovery was observed 2 to 8 weeks after treatment. m-THPC produced an equivalent effect at a lower concentration and less light energy than bacteriochlorin [28].

In 1996, Egger et al. studied the tissue distribution of protoporphyrins in a dog model after the i.v. administration of delta-aminolevulinic acid [29]. The authors showed PS distribution in the plasma, tissues, and urine. The highest concentration was observed 7 to 10 h after administration in the liver, pancreas, and prostate. The highest urinary excretion was 2 to 4 h after administration.

In 1998, Xiao et al. compared the intravesical instillation of two PSs (Photofrin II and 5-ALA) with a systemic i.v. injection of both compounds in orthotopic and heterotopic rat-bladder tumor models [30]. In the intravenous PS injection group, after a distribution time of 4 h, the authors registered the protoporphyrin IX ratio of tumor to bladder mucosa, submucosa, and muscle as 3:1, 5:1, and 8:1, respectively. With intravesical PS injection, fluorescence was detected only in the tumor and urothelium, with a tumor-to-bladder muscle ratio of 5:1 and loss of selectivity between the urothelium and tumor. After the i.v. injection of porpyrin, the main fluorescence came from the submucosa [30]. Bison et al. published another study in 1999 on the intravesical instillation of hematoporphyrin derivatives in a rat model of BC. They showed, that the best penetration of hematoporphyrin was achieved two hours after instillation. The authors also described an orthotopic BC animal model in which the bladder surface was ablated and AY-27 tumor cells were administered, which allowed superficial bladder tumors located in the bladder wall to be reached [31]. The above technique was described in detail in a later publication of the study group in 2002 [32].

In 2004, El Khatib et al. demonstrated for intravesical PDT with hexylester-5-ALA in the rat orthotopic bladder tumor model that the highest tumor-to-normal mucosa ratio was observed three hours after instillation (5.7 to 1). Tumor necrosis without damage to the intact urothelium was observed at a fluence of 20 J/cm^2^ at a concentration of 8 mM hALA, whereas the lower fluence had no effect and the higher fluence induced complete wall necrosis [33].

Berrahmoune et al. investigated the effects of PDT with protoporphyrin IX in a rat model in an adjuvant setting, mimicking the condition after fluorescence-guided TURBT. PDT significantly reduced the number of viable tumor cells injected into the bladder and the rate of cell implantation. The authors proposed a technique to reduce the recurrence rate after TURBT for non-muscle invasive BC [34].

In 2001, Zupko et al. published results of i.v. hypericin PDT in rats with subcutaneous heterotopic AY-27 tumors [35]. The authors emphasized the importance of the timing of the injection before PDT. An interval of 0.5 h before light exposure resulted in no tumor appearance within 10 days, whereas an interval of 6 h resulted in a 50% tumor regression. The authors concluded that PDT efficacy correlated with plasma concentration rather than tumor concentration, which may be a sign of the indirect vascular effects of hypericin versus direct cellular effects [35]. Kamuhabwa et al. studied the biodistribution of locally injected hypericin in an orthotopic rat-bladder tumor model. The authors showed that the uptake of hypericin occurs only through the tumor and normal urothelium, without fluorescence of the submucosa or bladder muscle. The tumor-to-normal bladder ratio was 12:1 four hours after exposure, and the authors emphasized this phenomenon as important for the selective PDT of urothelial bladder tumors [36]. Asanuma et al. examined the distribution and demonstrated the efficacy of i.v. chlorine-containing PS PAD-S31 in a rat orthotopic bladder tumor. The maximum tumor-to-normal ratio was reached 1.5–2 h after administration. The destructive effect was dependent on the light dose [37].

In 2019, Berndt-Paetz et al. published a study on PDT with tetrahydroporphyrin tetratosylate (THPTS) in an orthotopic rat model of muscle-invasive bladder cancer with AY-27 cells. The authors demonstrated an early onset of apoptosis leading to dose-dependent cytotoxicity. A single transurethral THPTS-PDT (100 μmol/L THPTS; 10 J/cm^2^) resulted in a significant reduction in the number of muscle-invasive tumors (2/10 versus 7/10 in the control group) and total tumor volume (60% reduction) 2 weeks after PDT [17]. 

Laranjo et al. recently described the luminescent Pt(II)-4,5,6,7-tetrahydropyrazolo[1,5-a]pyridine-fused chlorin as an effective PDT agent with tumor suppressive activity, no toxicity to healthy cells, and ideal properties as a luminescent probe, making it suitable for therapy monitoring and follow-up (so-called theranostics). The authors first compared different chlorins on different cell cultures (human melanoma, esophageal, and BC cells) in vitro and then investigated the PDT effect in a heterotopic mouse tumor model with melanoma cells. The authors suggested the described molecule as optimal [16]. In 2012, Miyazaki et al. introduced a new fiber probe for the homogeneous elimination of the bubble, called the homogeneous irradiation fiber probe, which provided a three-dimensional distribution of light. The author used the orthotopic rat BC model and PDT with Photofrin and showed significantly better placement of irritation, resulting in greater tumor reduction. The authors mentioned the advantage of the probe specifically in multifocal BC [38].

The above studies addressed the choice of PS, route of administration, distribution, dosing, and optimization of light conditions in various animal tumor models. Recent in vivo studies have investigated different combinations of PDT to enhance the effect of PDT. 

Korbelik et al. showed an additional beneficial effect of BCG for PDT with six different PSs in the heterotopic-bladder-tumor mouse model and recommended further clinical trials [39].

Bhuvaneswari et al. showed similar results for hypericin PDT in combination with bevacizumab [23]. Inoue et al. described in vitro and then in vivo that the addition of deferoxamine (inhibitor of ferrochelatase) to PDT with 5-ALA enhanced the apoptotic effect [40]. Gederas et al. demonstrated the enhancement of the therapeutic efficacy of intravesical bleomycin in combination with PDT with a novel PS TPCS2a^®^. The authors referred to the combination as “photochemical internalization” [41].

Several recent studies have examined combinations of vascular-targeted PDT with WST11 [42,43,44]. Corradi et al. showed very interesting results for the combination with anti-CTLA4 immunotherapy in a heterotopic urothelial carcinoma mouse model: significantly reduced tumor growth, reduced development of lung metastases, prolonged survival, and no tumor growth after tumor re-irradiation in pretreated mice [42]. Rosenzweig et al. studied vascular-targeted PDT with WST11 as a neoadjuvant treatment in a mouse model of UC 17 days before tumor resection. The study group demonstrated significant effects on local tumor shrinkage before surgery, reduction in local recurrence, and systemic progression after surgery, associated with prolongation of PFS and OS. The authors also demonstrated that the re-injection of mice more than 100 days after PDT did not result in tumor cell uptake, which is indicative of the induction of a long-term systemic immune response [43]. Alvim et al. recently published further data on vascular-targeted PDT (WST 11) in combination with a PD1 inhibitor and OX40 in a mouse model with allografted MB-49 upper tract urothelial carcinoma cells. The above combination therapy showed the best efficacy in inhibiting tumor growth and prolonging survival compared to the combination with only one of the above agents [44]. Poly(caprolactone) based biodegradable and nontoxic polymeric matrix was used to formulate an in situ thermo-responsive hydrogel to deliver doxorubicin [45]. In this study, doxorubicin and zinc phthalocyanine were loaded into an in situ thermo-responsive copolymer hydrogel at 37 °C. A tumor efficacy study in the 5637-cell xenograft model showed improved outcomes of combination therapy with delayed tumor growth and better survival.

The above studies provide an overview of the evidence on the effect of PDT in animal models. The properties of vascular targeted PDT in combination with modern oncologic drugs represent a very interesting prospect for future studies in the treatment of BC. Nevertheless, the limitations of the in vivo studies are mainly due to the difference in bladder models used compared to the human bladder. The difference in bladder size and wall thickness affects the diffusion of the drugs and illumination of the bladder [46].

### 3.5. Clinical Trials of PDT for Urothelial Carcinoma

Clinical evidence for PDT in urothelial carcinoma is currently limited to several series of patients with NMIBC [47,48,49,50,51,52,53,54,55,56,57,58,59,60,61], with most series using this therapy for carcinoma in situ (Cis) and others for multiple and recurrent papillary Ta/T1 disease. Table 1 summarizes the major series on the PDT of the urinary bladder.

Reported recurrence rates vary significantly between series when considering laser type, light delivery technique, light dosimetry (energy density), PS used and its dosage, type of non-muscle invasive bladder cancer (NMIBC) (papillary vs. cis, dimensions), and follow-up.

Overall, PDT appears to be a promising option for the treatment of cases of recurrent NMIBC that would otherwise undergo radical cystectomy. It can be combined with intravesical chemotherapy with mitomycin C, providing a more potent tumoricidal effect [55]. In the rare cases where the tumor is located in a bladder diverticulum, focal PDT can be used to eradicate the disease without risk of perforation [55]. PDT can also be repeated in patients who have received previous therapy or who are receiving radiotherapy or chemotherapy [58] without contraindication. 

An RCT compared the efficacy of BCG instillations (induction plus maintenance) with a single course of PDT with Photofrin in the treatment of patients at intermediate and high risks for non-muscle invasive bladder cancer [24]. A total of 124 patients were enrolled in the study. After intention-to-treat analysis and after as-treated analysis, the estimated median recurrence-free survival was 24.9 (BCG) versus 16.6 months (PDT) and 25.8 (BCG) versus 14.7 (PDT) months, respectively. The authors concluded that a single course of PDT with Photofrin was not superior to BCG maintenance therapy in this patient group. Conversely, the results of this study also could not rule out the superiority of BCG. Other authors have also pointed out that both side effects and economic considerations still favor BCG [54].

In addition, concerns about the relative complexity and side effects of bladder PDT still hinder its widespread clinical use [47,62]. Indeed, systemic (mainly skin photosensitivity) and local toxicity (bladder wall fibrosis/contracted bladder, vesicoureteral reflux, storage symptoms) are important problems to consider in treatment. 

These problems were most prevalent with early systemically administered PSs, which were slow to metabolize and had poor selectivity between tumor and detrusor, resulting in PDT-associated detrusor damage [63]. The intravesical administration of a PS (such as 5-ALA) is characterized by the selective uptake of the PS by healthy urothelium and TCC, with virtually no PSs entering the detrusor [64], making detrusor damage less likely. Because of the risk of pain and intravesical hyperthermia, PDT is traditionally performed under general or regional anesthesia. However, some authors have investigated the possibility of treatment under local anesthesia with promising results [68]. One study investigated the efficacy of PDT in four patients with extensive UC of the upper urinary tract [65] after oral administration of 5-ALA. The authors demonstrated complete remission in two patients and small residual papillary tumors on the distal ureter in two others, which were subsequently treated with a laser. Overall, the evidence on the clinical use of PDT for UC is limited to a few case series. In the majority of studies, porphyrins were used as PSs, and treatment was reported to be associated with higher recurrence rates compared with standard treatment.

Overall, there is obvious heterogeneity among the clinical studies, which makes it impossible to deliver a clear message on what photosensitizers are preferable for which conditions. Intravesical delivery seems more practical to avoid the systemic side effects. Future studies should include multiple arms to fairly compare among the photosensitizers and identify which subgroup of BC patients may benefit from PDT.

Recent advances in molecular technologies have led to the development of new emerging therapies in the oncological field, such as sonodynamic and chemodynamic therapy, also leading to the generation of ROS [66,67,68,69,70]. The available data also show the insufficient ability of the above-mentioned therapies to cure the tumor if used as a monotherapy. Thus, one way to improve the efficacy might be to combine the “dynamic” therapeutic modalities with each other and/or with traditional oncological therapies [66,67,68,69,70].

## 4. Summary and Conclusions

Photodynamic therapy for BC is performed by an intravesical or a systemic administration of a photosensitizing agent. The use of PDT in the bladder is of interest because of its relative ease of access via endoscopes and the high incidence of recurrence in UC of the bladder. Since the initial description of PDT in BC, numerous attempts have been made to use this form of therapy in routine clinical practice. The results presented in animal models have their limitations as bladder size and wall thickness affect drug diffusion and illumination exposure, but they still highlight the potential utilization of PDT in the neoadjuvant or adjuvant setting to reduce local recurrence rates after the endoscopic treatment of bladder cancer. In addition, the combination of PDT with intravesical chemotherapy, sono- or chemodynamic therapy, or with systemic therapy for improved local tumor control or integration of vascular-targeted PDT in combination with modern systemic drugs in order to boost local response in the urinary bladder could be a potential target for future clinical studies. New sensitizers with lower local toxicity and the development of devices that can deliver uniform dosage in a fractionated manner to the urinary bladder have the potential to reduce side effects and maximize tumor response rates, ideally in an outpatient setting.

## Figures and Tables

**Table 1:** Summary of clinical studies on PDT in urothelial carcinoma.

Author	Number of Patients	Indication	Treatment	Photosensitizing Agent	Route of Administration	Follow-Up (mo)	Side Effects	Recurrence (%)
Stenzl et al. [58]	6	Recurrent Cis after BCG (×5) + residual non-resectable papillary tumors (×1)	EMD + PDT	ALA	Intravesical	10–16	-Bladder spasms (×2) -Dysuria (×3)	1/6 (16.7)
Kriegmair et al. [59]	21	Recurrent and multifocal NMIBC	PDT	Synthetic porphyrin mixture	Intravenous	15–42	-Phototoxic erythema -Edematous swelling of the face (×5) -Dysuria + urgency (×19) -Bladder shrinkage with incontinence (×1)	6/12 (50) recurred at a mean follow-up of 11.5 months 9 patients → residual tumor at 3 months
Nseyo et al. [60]	36	Refractory Cis	PDT	Porfimer sodium	Intravenous	9–48	Bladder shrinkage (×7)	15/36 (42) at 3 mo 25/36 (69.5) at a mean f-up of 12 mo
Filonenko et al. [61]	45	NMIBC	TUR + simultaneous PDT	ALA	Intravesical	NR	NR	10/45 (22) at 12 mo
Uchibayashi et al. [62]	34	Refractory Cis	PDT	Hematoporphyrin derivative	Intravenous	NR	-Hematuria -Frequency -Skin photosensitivity -Bladder shrinkage	9/34 (26.5) at 3 mo 12/23 (52.2) at 1 yr 14/18 (77.8) at 2 yrs
Bader et al. [63]	17	Intermediate or high-risk NMIBC	PDT	HAL	Intravesical	6–21	-Bladder irritative symptoms (×15) -UTI (×5) -Macrohematuria (×1) -Serious AE (×4)	8/17 (47) at 6 mo 13/17 (76.5) at 9 mo 15/17 (88.2) at 21 mo
Nseyo [64]	58	NMIBC	Focal or whole-bladder PDT	Photofrin	Intravenous	NR	-Storage symptoms -Bladder shrinkage (22.2%) -Cutaneous photosensitivity (23.8%)	3/19 (15.8) for TaT1 papillary BCa 4/20 (20) for Cis 17/19 (89.5) for pts receiving prophylaxis of recurrence
D’Hallewin et al. [65]	15	Multifocal Cis	Whole-bladder PDT	Photofrin II	Intravenous	37	Bladder shrinkage (3/15)	6/15 (40)
Waidelich et al. [66]	4	Widespread UTUC	UUT PDT	5-ALA	Oral	24	-Nausea + emesis (×1) -Hypotension + tachycardia (×3)	2/4 (50)
Skyrme et al. [67]	24	NMIBC	MMC + PDT	5-ALA	Intravesical	24–33	-Macrohematuria -Storage symptoms -UTI	11/24 (45.8) at 24 mo
Shackley et al. [68]	19	NMIBC	PDT in local anesthesia	5-ALA	Intravesical	NR	-Painful bladder spasms -Storage symptoms	4/14 (28.6)
Waidelich et al. [69]	12	NMIBC	PDT	5-ALA	Intravesical	3–25	Storage symptoms	6/11 (54.5)
Kato et al. [70]	4	Cis	PDT	Photofrin	Intravenous	NR	-Photosensitivity -Transient increase in GOT and GPT	2/4 (50)
Walther et al. [60]	20	NMIBC	PDT	Photofrin II	Intravenous	23–56	-Storage symptoms -Vesicoureteral reflux -Bladder shrinkage -Pedal edema -Photosensitivity -Febbrile UTI	16/20 (80)
Jocham et al. [61]	15	NMIBC	PDT	DHE/HpD	Intravenous	24–54	Bladder shrinkage (×1) requiring cystectomy	6/15 (40)
Prout et al. [24]	20	NMIBC	PDT	Photofrin II	Intravenous	3	-Storage symptoms -Phototoxicity	10/19 (53)
